# 11.3 CLINICAL AND NEUROBIOLOGICAL EFFECTS OF A CONTINUOUS AEROBIC ENDURANCE TRAINING IN MULTI-EPISODE SCHIZOPHRENIA PATIENTS

**DOI:** 10.1093/schbul/sby014.041

**Published:** 2018-04-01

**Authors:** Berend Malchow, Sergi Papiol, Daniel Keeser, Boris Rauchmann, Katriona Keller-Varady, Alkomiet Hasan, Andrea Schmitt, Peter Falkai

**Affiliations:** Ludwig Maximilian University, Munich

## Abstract

**Background:**

Structural and functional brain alterations as well as cognitive deficits are well-documented findings in schizophrenia patients. Cognitive impairments affect the long-term outcome of schizophrenia and are the main contributors to disability. Aerobic endurance training has been shown to have effects on brain plasticity, gray and white matter volume as well as functional connectivity measures and on cognitive functioning in animal models and healthy humans. However, effects of physical exercise in combination in combination with cognitive remediation (CR) are unknown in schizophrenia.

**Methods:**

21 chronic schizophrenia patients and 21 age- and gender-matched healthy controls underwent 3 months of aerobic exercise (endurance training, 30 min, 3 times per week). 21 additionally recruited schizophrenia patients played table soccer (known as “foosball” in the USA) over the same period. After 6 weeks of endurance training or table soccer, all participants commenced standardized cognitive training with a computer-assisted training program. Clinical symptoms, thorough neuropsychological testing and multimodal neuroimaging with 3D-volumetric T1-weighted sequences, DTI and magnetic resonance spectroscopy (MRS) were performed on a 3T MR scanner at baseline and after the 3-month intervention and 3 additional training-free months. DNA from all subjects was genotyped with the Infinium PsychArray Chip (Illumina, San Diego, CA, USA). Polygenic risk scores were calculated and associated with hippocampal subfield volume change.

**Results:**

In summary, a 3-month endurance training program combined with CR therapy for the last 6 weeks of the intervention period was feasible (Keller-Varady et al. 2016) and had positive effects on everyday functioning in multi-episode schizophrenia patients. Deficits improved from medium to mild as assessed with the GAF. Negative symptoms, short- and long-term verbal memory and cognitive flexibility also improved with endurance and cognitive training (Malchow et al. 2015). We could demonstrate grey matter volume increase in the left temporal lobe in schizophrenia patients undergoing endurance training. A non-endurance and coordinative training stimulus like playing table soccer led to a clearly distinct pattern of grey matter alterations in schizophrenia patients (Malchow et al. 2016). There were no effects of the intervention on structural and functional brain networks in schizophrenia patients as well as MRS measures (in preparation). No effects of PRSs were found on total hippocampal volume change. Subfield analyses showed that the volume changes between baseline and 3 months in the left CA4/DG were significantly influenced by PRSs in schizophrenia patients performing aerobic exercise. A larger genetic risk burden was associated with a less pronounced volume increase or a decrease in volume over the course of the exercise intervention. Results of exploratory enrichment analyses reinforced the notion of genetic risk factors modulating biological processes tightly related to synaptic ion channel activity, calcium signaling, glutamate signaling and regulation of cell morphogenesis (Papiol et al. 2017).

**Discussion:**

Exercise interventions are feasible and effective interventions for people with schizophrenia and might also help to disentangle the underlying brain pathology of the disorder.

